# Healthy weight in childhood

**DOI:** 10.2471/BLT.22.289049

**Published:** 2023-02-08

**Authors:** Oliver Huse, Tim Lobstein, Jo Jewell, Sarah Zahr, D’Arcy Williams, Karimen Leon, Fiona Watson

**Affiliations:** a United Nations Children’s Fund East Asia and Pacific Regional Office, 19 Phra Athit Rd, Phra Nakhon, Bangkok 10200, Thailand.; b Policy Section, World Obesity Federation, London, England.; c Nutrition and Child Development Section, United Nations Children’s Fund, New York, United States of America.; d Latin America and the Caribbean Regional Office, United Nations Children’s Fund, Panama, Panama.

Nearly one fifth of the world’s children are experiencing overweight or obesity – that is, an estimated 39 million children younger than five years and 340 million children aged 5–19 years.^1^ The toll of unhealthy diets and overweight in terms of health, social and financial costs are significant, representing almost an estimated 3% of global gross domestic product – a similar economic impact to that of smoking or armed violence, war and terrorism.^2^

Many governments in low- and middle-income countries continue to grapple with undernutrition and micronutrient deficiencies in addition to sharp increases in overweight among children. This triple burden of malnutrition has intensified as these countries face new challenges including the aftermath of the coronavirus disease 2019 pandemic, climate change and global economic recession.^3^ One consequence has been a substantial rise in the price of fresh foods which, combined with increased sales and marketing of ultra-processed foods, are driving all forms of malnutrition including overweight.^4^ Ultra-processed foods are those that contain little or no whole food, are palatable, energy-dense and low in essential nutrients, such as snacks and ready meals. 

A set of promising food policies are showing positive impact in reducing the purchase and consumption of ultra-processed foods in some high- and middle-income countries, and are recommended as part of a suite of measures to address overweight and obesity by the World Health Organization (WHO).^5^ The most effective measures include the restriction of children’s exposure to the marketing of food and non-alcoholic beverages based on robust nutrient profiling schemes that clearly define healthier and less healthy foods and beverages. Measures also include fiscal policies such as taxes and industry levies directed at snack foods and sugar-sweetened beverages, mandatory front of package warning labels and improving school food environments. The World Health Assembly recently endorsed a Global Acceleration Plan to Stop Obesity designed to scale up action and demonstrate impact in a set of frontrunner countries.^6^

The United Nations Children’s Fund (UNICEF) included these key policies on prevention of childhood overweight in its Strategic Plan 2018–2021, and has developed programming guidance for its regional and country offices to scale up work and support global norms and guidelines. Subsequently, the prevention of overweight was highlighted as a priority in the UNICEF Nutrition Strategy 2020–2030.^7^ United Nations agencies, including UNICEF, are important partners to national governments in designing response strategies.

UNICEF is in a unique position to support governments through its on-the-ground presence in 130 countries, child rights mandate and long-standing role as a trusted adviser to national governments. Furthermore, UNICEF, together with WHO, has long experience in guiding countries in implementing legislation on the marketing of breastmilk substitutes, and is now scaling up its legal support to countries on food policies for overweight and obesity prevention.^8^ UNICEF’s experience in promoting children’s rights legislation can help governments to develop a robust and comprehensive regulatory framework on policy implementation for prevention of overweight in children. In addition, UNICEF supports high-quality evidence generation and is well positioned to mobilize resources to support governments.

Here we bring the experience of UNICEF in stimulating government response to the growing challenge of overweight among children in low- and middle-income countries through the application of a landscape analysis tool. UNICEF in consultation with WHO developed this tool, which has been piloted in nine countries (China, Costa Rica, India, Indonesia, Mongolia, Peru, Philippines, United Republic of Tanzania and Viet Nam).^9^ The tool is designed to capture a comprehensive picture of childhood overweight and obesity by describing prevalence and trends, analysing key risk factors in early and later childhood, and assessing policies and programmes to address overweight and obesity. The tool supports the collation of quantitative data and collection of qualitative information through key informant interviews and multisectoral validation workshops, with the main aim of identifying a prioritized set of feasible policy actions for preventing childhood overweight and obesity within a particular country context.

## Positioning overweight prevention

The landscape analysis tool provides an opportunity to position overweight within the broader malnutrition agenda. Traditionally, interventions in low- and middle-income countries have concentrated on addressing undernutrition in children younger than five years. The landscape analysis brings the growing challenge of overweight to the forefront with a focus on all children, including older children and adolescents, among whom prevalence tends to be higher. The data challenge the belief that overweight is an exclusive problem of the wealthy, showing how overweight is increasing in low- and middle-income countries and affecting children in lower wealth groups and rural areas. The face of malnutrition is changing as the triple burden of malnutrition becomes the new norm. The application of the landscape analysis reinforces the conclusion that the prevention of overweight and obesity must be integrated into the response to child malnutrition in low- and middle-income countries.

## Tackling the drivers of overweight

Children’s dietary and health behaviour is determined by the environment in which the children live.^10^ The landscape analysis tool collates data on the risk factors for overweight and illustrates how the environment provides many incentives to gain weight, from increasing availability, affordability and promotion of cheap ultra-processed foods, to reduced opportunities for outdoor play and increased sedentary, screen-watching time. Landscape analysis switches attention away from individual responsibility, and instead focuses on environmental causes and the role of multiple systems in influencing nutrition outcomes. This shift also avoids stigmatizing children who are experiencing overweight or obesity. These children are likely to have lifelong physical consequences to their growth and health, and are often blamed for their problem and experience significant discrimination and bullying.^11^ Attempts to change behaviour through educational means are likely to be frustrated as they fail to address the external influences driving overweight and obesity. Rather, the food, social protection, education, water and sanitation, and health systems all have a role to play in addressing overweight with population-level measures, including regulating the practices of the food and beverage industry.

## Gathering stakeholders and sectors 

Through the validation workshop, multiple sectors and stakeholders within government and development partners are brought together to address childhood overweight and obesity. In some low- and middle-income countries, this workshop may be the first time that stakeholders come together to consider a multisectoral response to childhood overweight and obesity. Such response involves considering actions in systems beyond the health sector – the traditional sector tackling overweight in many low- and middle-income countries. The workshop also brings different agencies with a range of skills to lend their expertise in planning and implementation of polices to prevent overweight, including agencies with authority to regulate foods and food and beverage industry practices. The validation workshops can facilitate the establishment of formal governmental mechanisms to address overweight and obesity as well as less formal working groups to focus on specific policy actions.

## Supporting governments to act 

The overall purpose of the landscape analysis is to prioritize feasible policy actions that consider the rising prevalence of overweight as well as the existing challenges of undernutrition and micronutrient deficiencies. The landscape analysis tool encourages users to consider the existence and coverage of policy and programme approaches to tackle all forms of malnutrition. Such double-duty approaches require a broad systems approach with a focus on early prevention and addressing the availability, affordability, acceptability and appeal of nutritious diets for women and children, as well as a focus on access to quality nutrition services and optimal caregiver practices.^12^

However, additional influences that increase the risk of weight gain in children exist, and they might not be addressed in the double-duty policy approach. Examples include access to safe and attractive facilities for physical activity, and reducing time spent in sedentary behaviour.^13^ Furthermore, the tool encourages users to consider the wide range of commercial and political factors, especially linked to the products, policies and practices of the food and beverage industry, that strongly influence the drivers of obesity, the public discourse around overweight, as well as the policy and programmatic decision-making process in low- and middle-income countries related to food policies.

The landscape analysis tool can help to harness political will and facilitate consensus on feasible priorities for action to address overweight and obesity. The validation workshops can support the development of action plans and national strategies to address overweight and obesity, as well as advance specific policies such as front-of-package nutrition labelling, sugar-sweetened beverage taxation, and unhealthy food and beverage marketing restrictions. The challenge of addressing childhood overweight at the same time as undernutrition and micronutrient deficiencies requires additional capacity and resources that may not be readily available in resource-poor settings. The landscape analysis helps to identify the barriers for effective action, and define the information and capacity gaps so that resources can be sought.

## Conclusions

Overweight in children is an issue of child rights. Global mandates, including the sustainable development goals, the Rome Declaration on Nutrition, the United Nations Decade of Action on Nutrition 2016–2025, United Nations General Assembly and World Health Assembly declarations and outcome documents on the Prevention and Control of Non-Communicable Diseases, and the Commission on Ending Childhood Obesity support the need to prioritize action to prevent overweight and obesity in children. The landscape analysis represents a practical tool to support governments to action these declarations to which many low- and middle-income countries have signed up. Low- and middle-income countries that have conducted landscape analyses have produced reports and policy briefs to guide further actions. These countries are now focusing on introducing and strengthening policies and programmes based on the outcomes of the landscape analysis and agreement on priority actions. More countries are implementing landscape analyses using the tool to guide and focus their efforts to prevent overweight and obesity among children.
